# Editorial: Airway remodeling in asthma—what is new?

**DOI:** 10.3389/falgy.2023.1129840

**Published:** 2023-04-05

**Authors:** Akira Yamasaki

**Affiliations:** The Department of Respiratory Medicine and Rheumatology, the Division of Multidisciplinary Internal Medicine, Faculty of Medicine, Tottori University, Yonago, Japan

**Keywords:** asthmatic airway remodeling, 3D culture model, Drosophila melanogaster model, Th17, gender differences

**Editorial on the Research Topic** Airway remodeling in asthma—what is new?

## Introduction

Asthma, a chronic inflammatory disease that affects the airways, is characterized by variable degrees of airflow obstruction, recurrent wheezing, shortness of breath, tightness in the chest, chronic cough, and sputum production. Severe asthma is defined as a condition (a) requires treatment with high-dose inhaled corticosteroids plus a second controller during the full previous year and/or systemic corticosteroids during 50% or more of the previous year to prevent symptoms from becoming “uncontrolled”, or (b) remains “uncontrolled” despite this therapy (1). Severe asthma is associated with airway inflammation and remodeling (2).

The pathological features of airway remodeling include epithelial cell shedding, mucous metaplasia, submucosal inflammatory cell infiltration, increased airway smooth muscle (ASM) mass, and extracellular matrix (ECM) deposition. Eosinophils are important inflammatory cells in the pathogenesis of asthma-associated airway remodeling because they produce transforming growth factor-*β* (TGF-*β*), which is an important mediator of airway remodeling. Moreover, neutrophilic inflammation increases the levels of inflammatory mediators, such as LTB4, IL-8, IL-17, and TNF-*α*, which play a role in the pathophysiology of airway remodeling by inducing ASM cell proliferation and migration and upregulating MUC5A and MUC5B expression in epithelial cells (3). Airway inflammation is often proposed as an upstream driver of remodeling and therefore an appropriate target to prevent detrimental structural changes, although it is important to appreciate that such events can occur independent of inflammatory stimuli. Airway remodeling has been observed in short-term airway constriction without airway inflammation (4) and in preschool children with severe recurrent-wheeze childhood asthma that is not associated with airway inflammation (5).

The pathophysiology of airway remodeling is complex. Additionally, the high death rate of older patients with asthma poses a significant therapeutic challenge. Upon aging, pulmonary function reduction and residual volume increase are observed, with a more evident decline in pulmonary function in patients suffering from asthma (6). Aghahi et al. reported greater airway fibrosis in older individuals with asthma compared with control groups consisting of young and older individuals (7). However, thickening of the smooth muscles of airways is not related to the duration of asthma (8, 9). Therefore, the natural course of airway remodeling from the infant to the geriatric stages has not been fully elucidated. Furthermore, no single drug can reduce or reverse airway remodeling. Therefore, establishing multiple models and assessing airway remodeling using reliable biomarkers are a critical and burning issue in asthma treatment. In this Research Topic, numerous aspects of airway remodeling studies and updates on the pathogenesis of asthma and airway inflammation have been reported.

Ben Hamouda et al. studied both ultrastructural and genetic changes in ASM cells cultured on ECM using a novel 3D culture model in their original article. Since modified ECM profiles may affect the characteristics of ASM cells in asthma (10), their 3D culture model with a biological bronchial matrix presents a remarkable advantage for studies in an *in vivo* environment, compared to 2D culture models. Ben Hamouda et al. found that the number of ASM cells from control and asthmatic horses increased when these cells were cultured on recellularized bronchi from the control. They also detected the presence of cellular senescence and debris when asthmatic or control ASM cells were cultured on recellularized asthmatic bronchi. Targeted next-generation sequencing revealed that *AGC1*, *MYO10,* and *JAM3* expression levels were upregulated in control matrices/control cells compared to levels in asthmatic matrices/asthmatic cells, while *TAGLN* was overexpressed in asthmatic matrices/asthmatic cells. The authors concluded that the health status of the ECM contributes to genetic changes in ASM cells and possibly their survival.

Both *in vitro* and *in vivo* animal models have demonstrated the importance of airway epithelial cells in airway remodeling in asthma. Ehrhardt et al. reviewed a *Drosophila* model to investigate the role of the airway epithelium in asthmatic airway remodeling; the *Drosophila* airway is simple and is composed of a single layer of uniformly arranged epithelial cells. These authors examined genetic and environment-induced structural changes in the airway as well as the molecular aspects of such changes. Since *Drosophila* has a significantly shorter lifespan than humans and has conserved pathways and target genes, it is highly recommended for studies regarding airway epithelial cell biology, particularly in the early stages of airway remodeling, observed in preschool children.

Margelidon-Cozzolino et al. reviewed the role of the Th17 cytokines, IL-17 and IL-22, in airway remodeling and found that IL-17 levels were elevated in severe asthma and were associated with non-type 2 asthma, particularly neutrophilic asthma. IL-22 levels were also elevated in asthma and associated with *in vitro* airway remodeling. Biologics targeting IL-17 were not promising. The authors did, however, raise concerns about patients' eligibility and the possibility for testing IL-22-targeted therapies in a clinical trial. They demonstrated the importance of Th17 cytokines in the pathophysiology of asthma and proposed future research directions, including Th2 and Th17 cell exhaustion.

Regarding the role of airway-resident cells in airway remodeling, two interesting papers on mast cells and pericytes have been published. Although mast cells are important cells involved in type I hypersensitivity reactions owing to their infiltration into ASMs, their importance in airway hyperresponsiveness and remodeling has only been recognized in asthma and not in eosinophilic bronchitis (11, 12).

Alzahrani et al. demonstrated that a medium from FcɛRI-activated mast cells induces steroid insensitivity in ASM cells by altering their transactivation properties in their original article. This steroid insensitivity is caused by a decrease in glucocorticoid receptor *α* (GR*α*) transactivation. This study demonstrates a novel therapeutic approach for severe asthma *via* reorganization of interactions between mast cells and ASM cells.

Myofibroblasts also play an important role in the pathophysiology of asthmatic airway remodeling. Myofibroblasts are derived from fibroblasts, fibrocytes, epithelial cells, ASM cells, and pericytes (12). Bignold et al. demonstrated TGF-*β*− or IL-13-induced pericyte migration and periostin synthesis in their original article. Furthermore, they elucidated the signaling mechanisms involving periostin production and pericyte migration *via* IL-13 and TGF-*β*. The present study confirms the importance of pericytes and profibrotic cytokines in asthmatic airway remodeling.

Ekpruke and Silveyra reviewed the factors influencing the gender-associated differences in airway inflammation and remodeling. They summarized the differences in the structure and function of the respiratory system, immune responses, and hormonal balance, including those of estrogen, progesterone, testosterone, and leptin. They also reviewed and summarized the genomic framework and gender-specific factors, such as social lifestyle and occupation, thereby presenting a novel aspect of airway remodeling in asthma based on gender differences. The prevalence of and mortality due to asthma are higher in women than in men; thus, gender-associated differences should be considered when addressing asthma mortality in elderly patients, particularly female patients.

In conclusion, the areas of scientific focus in this Research Topic shed light on several aspects of airway remodeling in asthma; the findings from three excellent review articles and three original articles provide insights into a wide variety of aspects regarding asthma-associated airway remodeling. Future studies of airway remodeling should focus on clarifying the complexity and interaction of airway inflammatory cells, structural cells, and the ECM by using an *in vivo* model with potential drugs to reduce and reverse airway remodeling. Furthermore, it is critical to consider the nature of airway remodeling as well as how and when it occurs, persists, or resolves. The establishment of reliable biomarkers is also important to study airway remodeling in human asthma.
